# Perfusion Parameter Map Generation from 3 Phases of Computed Tomography Perfusion in Stroke Using Generative Adversarial Networks

**DOI:** 10.34133/research.0689

**Published:** 2025-04-30

**Authors:** Cuidie Zeng, Xiaoling Wu, Fusheng Ouyang, Baoliang Guo, Xiao Zhang, Jianghua Ma, Dong Zeng, Bin Zhang

**Affiliations:** ^1^ School of Biomedical Engineering, Southern Medical University, Guangzhou, Guangdong, China.; ^2^ Department of Radiology, The First Affiliated Hospital of Jinan University, Guangzhou, Guangdong, China.; ^3^ Department of Radiology, The Eighth Affiliated Hospital of Southern Medical University (The First People’s Hospital of Shunde Foshan), Foshan, China.; ^4^ Medical AI Lab, The First Hospital of Hebei Medical University, Hebei Medical University, Shijiazhuang, Hebei, China.; ^5^ Hebei Provincial Engineering Research Center for AI-Based Cancer Treatment Decision-Making, The First Hospital of Hebei Medical University, Hebei Medical University, Shijiazhuang, Hebei, China.; ^6^ Department of Oncology, The First Hospital of Hebei Medical University, Hebei Medical University, Shijiazhuang, Hebei, China.; ^7^ School of Life Science and Technology, Xi’an Jiao Tong University, Shaanxi, China.

## Abstract

Computed tomography perfusion (CTP) plays a crucial role in guiding reperfusion therapy and patient selection for acute ischemic stroke (AIS) through perfusion parameter maps of the brain; however, its widespread use is limited by the complexity of acquisition protocols and high radiation dose. Previous studies have attempted to reduce radiation exposure by equally lowering the temporal sampling rate; however, it may miss the peak of arterial enhancement, leading to underestimation of blood flow parameter. Here, we investigate the feasibility of using a generative adversarial network (GAN) to generate perfusion maps from 3 phases of CTP (mCTP). The three phases were chosen based on the multiphase computed tomography angiography scanning protocol: the peak arterial input function phase, the peak venous output function phase, and the delayed venous output function phase. The findings demonstrate that the GAN model achieved high visual overlap and performance for cerebral blood flow and time-to-maximum maps, with a mean structural similarity index measure of 0.921 to 0.971 and 0.817 to 0.883, a mean normalized root mean squared error of 0.019 to 0.108 and 0.058 to 0.064, and a mean learned perceptual image patch similarity of 0.039 to 0.088 and 0.141 to 0.146, respectively. For the 2 external datasets, the volume agreement between the model- and CTP-derived infarct and hypoperfusion areas was the intraclass correlation coefficient of 0.731 to 0.883 and 0.499 to 0.635, and the Spearman correlation coefficient of 0.720 to 0.808 and 0.533 to 0.6540, respectively. Qualitative assessments of diagnostic quality further confirmed that the mCTP-derived maps were comparable to those obtained from traditional CTP. In conclusion, the GAN-based model is effective in generating perfusion maps from mCTP, which could serve as a viable alternative to traditional CTP in the diagnostic evaluation of AIS.

## Introduction

Acute ischemic stroke (AIS) is caused by thrombotic or embolic occlusion of cerebral arteries, resulting in damage to brain cells [1]. AIS is a leading cause of death and is a major cause of serious disability worldwide [2]. Time-to-treatment is critical in the management of AIS, as the time from symptom onset to recanalization decreases the likelihood of a favorable clinical outcome [3].

Computed tomography perfusion (CTP) plays a crucial role in quantifying the volume of infarct and hypoperfusion areas through perfusion maps, including cerebral blood flow (CBF), cerebral blood volume (CBV), mean transit time (MTT), and time to maximum (*T*_max_) [4]. However, CTP imaging has several drawbacks, such as higher radiation exposure to the patients, with a total dose of 5,260 mGy-cm for CTP versus 3,222.3 mGy-cm for CT angiography (CTA) [5]. In addition, CTP can be time-consuming to acquire, process, and interpret, and it has a relatively high rate of postprocessing failures, which may affect its diagnostic accuracy during the critical time window [6–8]. There also exist studies that reduce the temporal resolution by sampling at equal intervals, thereby realizing a reduction in radiation dose of CTP [9,10]. However, they have low possibility of clinical realization and reproducibility.

As an alternative, multiphase CTA (mCTA) is used to assess the collateral circulation [11,12], consisting of three phases of CTA with sampling times as follows: the peak of arterial input function (AIF), the peak time of venous output function (VOF), and the delay time from peak time of VOF. Three-phase mCTA can be regarded as a super-low temporal resolution CTP, as it also captures information on blood flow changes similar to CTP. Thus, mCTA has the potential to estimate perfusion maps through machine learning techniques [13,14], showing the capability of mCTA in providing quantitative information. However, these machine learning methods might underestimate perfusion maps due to the limited frames in mCTA and their sensitivity to hyperparameters in deconvolution algorithms. Moreover, mCTA generally involves higher radiation doses compared to CTP in three phase calculations, with a total dose of 60 mGy versus 25 mGy (measured on the same phantom) for the eye [11], and a higher volume of contrast agent injected, such as 80 ml versus 40 ml, when using the same contrast material, injection rate, and saline chase [15]. The administration of contrast media can also increase the risk of renal injury, particularly in patients with preexisting renal issues, who are at a higher risk of post-contrast acute kidney injury [6]. To this end, we propose ultralow temporal resolution CTP, referred to as multiphase CTP (mCTP), which can be acquired using acquisition timings similar to the three-phase mCTA (i.e., the phases controlled by the AIF and VOF functions), thereby reducing the high radiation dose related to CTP imaging.

**Fig. 1.**
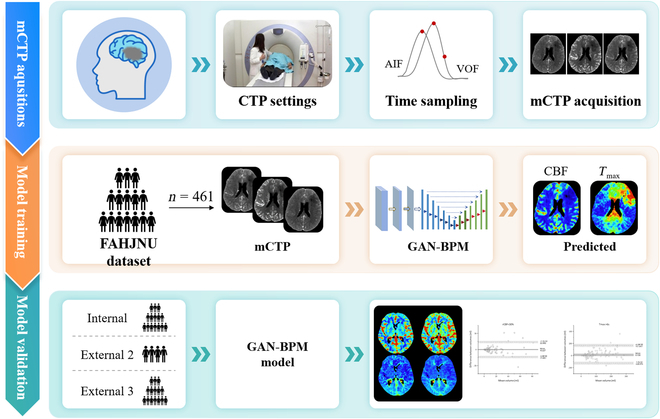
Overview of the study design.

To our knowledge, this is the first study to investigate the feasibility of generating perfusion maps from mCTP using deep learning, without relying on either deconvolution or non-deconvolution techniques. To predict CBF and *T*_max_ perfusion maps, we propose a generative adversarial network-based brain perfusion map generation (GAN-BPM) model. The GAN-BPM model leverages GANs [16], which are highly effective for translating between different image modalities [17,18]. We evaluated the correlation between the predicted and actual parameter maps, and the ability of the model to accurately identify core and hypoperfusion regions (Fig. 1).

## Results

### Baseline characteristics

The First Affiliated Hospital of Jinan University (FAHJNU) dataset, used to develop the GAN-BPM model for predicting perfusion maps, included 563 patients with a total of 8,445 slices. Demographic data, onset-to-examination time, and lesion volumes for the patients from FAHJNU, First Affiliated Hospital of Xi’an Jiaotong University (FAHXJU), and Shunde Hospital of Southern Medical University (SHSMU) datasets are listed in Table 1. From the ISLES2018 external dataset, 486 slices had follow-up diffusion-weighted imaging (DWI) images within 3 h, but only 287 slices had core masks manually segmented by experts and were used for external testing.

**Table 1.** Patient characteristics of 4 datasets

Variables	FAHJNU	FAHXJU	SHSMU
Cases, *n*	563	48	115
Gender, *n* (%) female	270 (47.9)	18 (37.5)	38 (33.0)
Median age, years (IQR)	69 (58, 77)	63 (56, 70)	64 (56, 72)
Median OET, h (IQR)	7 (2, 24)	10 (8, 12)	52 (56, 72)
Median HV, ml (IQR)	38.8 (22.5, 55.1)	142.7 (102.2, 209.8)	373.6 (314.0, 436.3)
Median CV, ml (IQR)	23.8 (9.8, 38.2)	19.5 (11.3, 30.7)	39.8 (33.1, 46.0)
Median MR (IQR)	1.7 (1.0–3.5)	7.5 (5.1, 9.7)	9.7 (8.2, 11.7)
ISLES2018
Follow-up DWI slices (%)	287 (59.1)		

IQR, interquartile range; OET, onset-to-examination time; HV, hypoperfusion volumes; CV, core volumes; MR, mismatch ratio

### Predictive performance of the GAN-BPM model

**Fig. 2.**
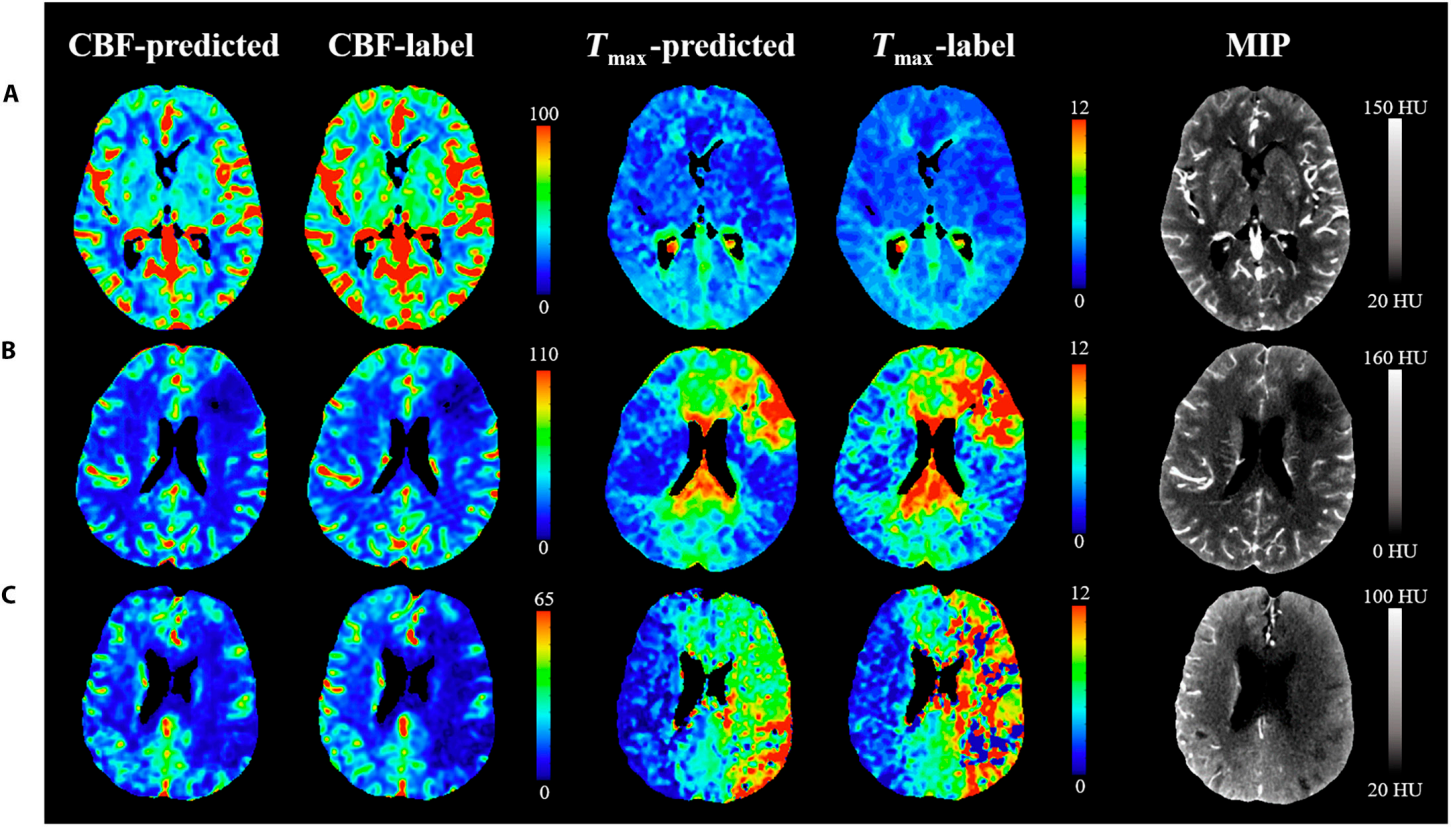
CBF and *T*_max_ maps derived from the model and CTP for three patients with different core and hypoperfusion volumes in the test dataset, along with the corresponding MIP maps. Perfusion maps are displayed in pseudo-color, with red representing higher attention. MIP refers to the maximum intensity projection images generated from the mCTP. (A) A 76-year-old female with a 12-h onset-to-examination time; the total core and hypoperfusion volumes were 15.4 and 7.8 ml, respectively, with a mismatch ratio of 0.5. The model predicted total core and hypoperfusion volumes of 15.1 and 10.3 ml with a mismatch ratio of 0.7. (B) A 59-year-old male with a 2-h onset-to-examination time; the total core and hypoperfusion volumes were 43.8 and 230.1 ml, respectively, with a mismatch ratio of 5.3. The model predicted total core and hypoperfusion volumes of 34.0 and 157.9 ml, respectively, with a mismatch ratio of 4.6. (C) An 86-year-old male with a half-hour onset-to-examination time; the total core and hypoperfusion volumes were 174.5 and 289.9 ml, respectively, with a mismatch ratio of 1.7. The model predicted total core and hypoperfusion volumes of 172.6 and 177.8 ml, respectively, with a mismatch ratio of 1.0. The model exhibited lower accuracy with larger hypoperfusion tissue sizes.

**Fig. 3.**
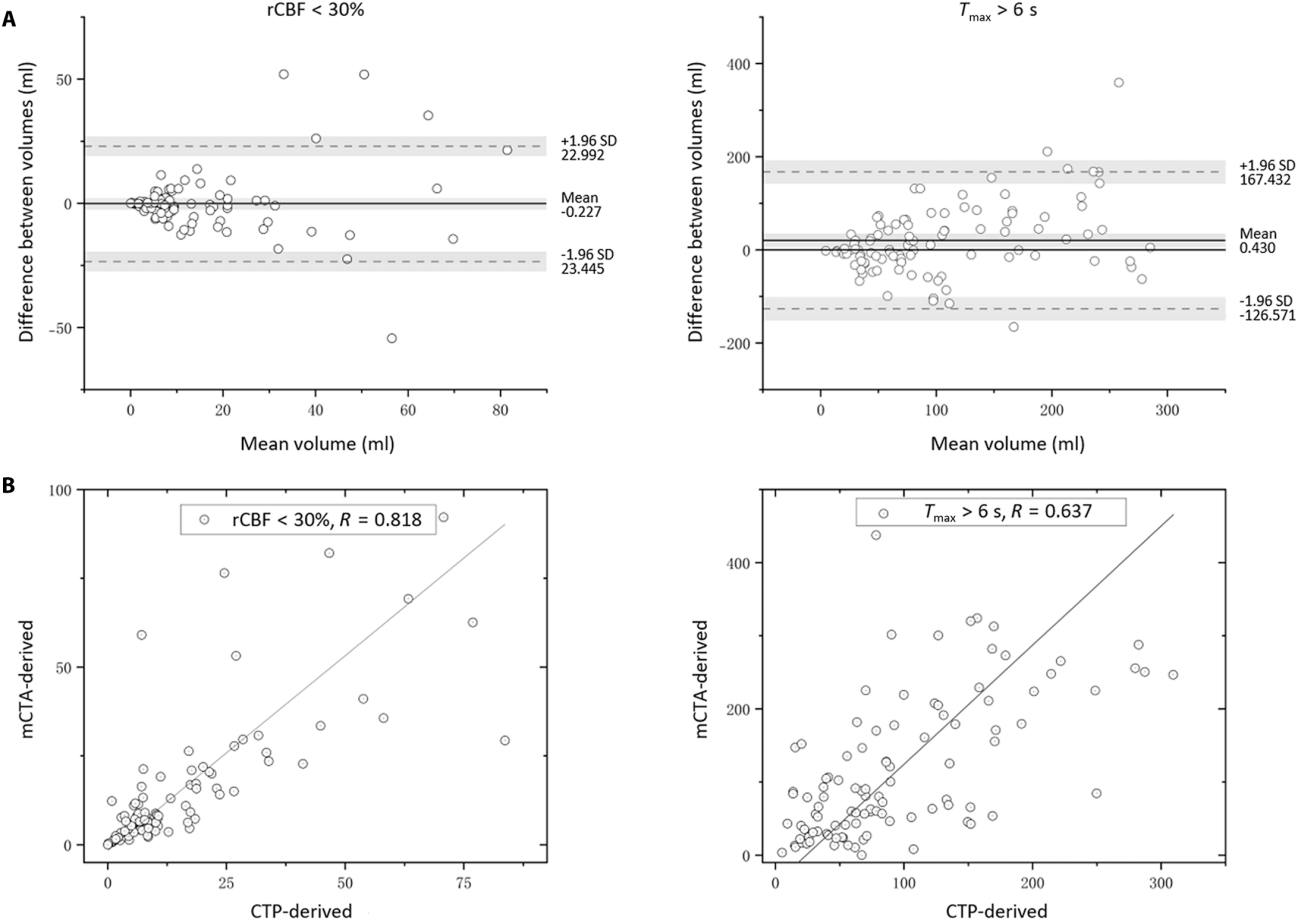
Bland–Altman and scatter plots for core and hypoperfusion volumes derived from the model and CTP standard reference in the testing dataset. (A) Bland–Altman plots showing core tissue (left) and hypoperfusion tissue (right) volumes from 110 patients. (B) Scatter plots with SCC for core tissue (left) and hypoperfusion tissue (right) volumes from 110 patients, illustrating the relationship between the model and the reference.

Figure 2 illustrates the CBF and *T*_max_ maps generated by both the predicted model and the deconvolution algorithm, based on CTP series images from three patients with varying core and hypoperfusion volume sizes in the test dataset. Visual inspection revealed substantial similarities between the predicted values and those derived from CTP for both CBF and *T*_max_. Compared to the maximum intensity projection (MIP) images produced from mCTP, the predicted CBF images accurately captured the vascular distribution and the presence of occlusions. Furthermore, the Dice coefficients for core and hypoperfusion regions across the three cases were 0.994 and 0.536, 0.931 and 0.862, and 0.910 and 0.657, respectively. In terms of quantitative evaluation, the model achieved excellent performance, with a mean ± SD of normalized root mean squared error (NRMSE) 0.021 ± 0.001, structural similarity index measure (SSIM) 0.959 ± 0.002, and learned perceptual image patch similarity (LPIPS) 0.059 ± 0.003 for the CBF-predicted model, and NRMSE 0.046 ± 0.002, SSIM 0.920 ± 0.001, and LPIPS 0.098 ± 0.002 for the *T*_max_-predicted model. The Bland–Altman plot shown in Fig. 3A presents the agreement between the spatial core (segmented using a threshold of rCBF < 30%) and hypoperfusion tissue volumes (segmented using a threshold of *T*_max_ > 6 s) from the mCTP-predicted and CTP-derived images across 102 patients in the full testing dataset. The mean volume difference for the core, defined by rCBF < 30%, between the mCTP-derived (median, 7.1 ml; interquartile range [IQR], 3.9 to 16.8) and CTP-derived (median, 7.6 ml; IQR, 4.4 to 17.6) was −0.2 ml (limits of agreement [LoA], −23.4 to 23.0). The mean volume difference for the hypoperfusion, defined by *T*_max_ > 6 s, between the mCTP-derived (median, 80.2 ml; IQR, 41.9 to 178.7) and CTP-derived (median, 72.5 ml; IQR, 41.0 to 134.2) was 0.4 ml (LoA, −126.6 to 167.4). Volume consistency, as shown in Table 2, was good for core lesions and moderate for hypoperfusion lesions. The scatter plots in Fig. 3B demonstrate the correlation between the mCTP- and CTP-derived volumes, with a Spearman correlation coefficient (SCC) of 0.818 for core volumes and 0.637 for hypoperfusion volumes (both *P* < 0.050).

**Table 2.** Quantitative metrics and volumes agreement of 5 types of CTP phase compositions. Pre-*x* means that advance *x* frame from the arterial function peak frame. Late-*x* indicates that delay *x* frame from the arterial function peak frame. All the quantitative indexes were represented as mean ± SD. Volume agreement was assessed using the ICC and SCC. This was tested on the total volumes for each patient, based on 15 slices (for most patients) or fewer.

	Metrics	Pre-2	Pre-1	Peak	Late-1	Late-2
CBF	NRMSE	0.020±0.001	0.019±0.001	0.021±0.001	0.029±0.001	0.027±0.001
	SSIM	0.955±0.02	0.958±0.002	0.959±0.002	0.952±0.002	0.953±0.002
	LPIPS	0.067±0.004	0.062±0.040	0.059±0.003	0.06±0.0030	0.063±0.003
*T*_max_	NRMSE	0.054±0.003	0.049±0.002	0.046±0.002	0.046±0.001	0.049±0.002
	SSIM	0.912±0.001	0.919±0.001	0.920±0.001	0.922±0.001	0.917±0.001
	LPIPS	0.102±0.002	0.097±0.002	0.098±0.002	0.097±0.002	0.099±0.002
rCBF < 30%	SCC	0.735	0.792	0.818	0.809	0.741
	ICC	0.672 (0.550, 0.766)	0.777 (0.687, 0.844)	0.771 (0.680, 0.839)	0.811 (0.733, 0.869)	0.731 (0.626, 0.810)
*T*_max_ > 6 s	SCC	0.311	0.473	0.637	0.612	0.534
	ICC	0.327 (0.142, 0.489)	0.480 (0.316, 0.616)	0.630 (0.506, 0.729)	0.573 (0.426, 0.690)	0.481 (0.317, 0.618)

NRMSE, normalized root mean squared error; SSIM, structural similarity index measure; LPIPS, learned perceptual image patch similarity; ICC, intraclass correlation coefficient; SCC, Spearman correlation coefficient

### Model performance across different phase compositions

**Fig. 4.**
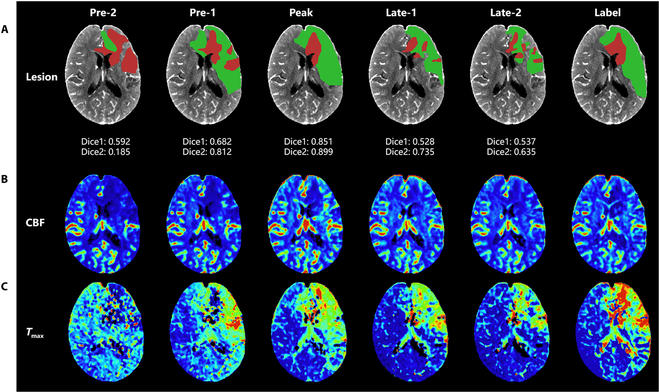
Segmented lesion regions, CBF maps, and *T*_max_ maps for 5 phase compositions derived from the model, compared with the CTP standard reference, from a patient with moderate infarct size and extensive hypoperfusion size. Dice1, Dice coefficient for core region (in red); Dice2, Dice coefficient for hypoperfusion region (in green). Columns 1 to 5 show the results of 5 types of mCTP phase compositions; time intervals of advanced or delayed from the peak frames were 2 s for Pre-1 and Late-1 and 4 s for Pre-2 and Late-2; column 6 displays the standard reference from CTP. This case involves a 48-year-old male with a 12-h onset-to-examination time, and total core and hypoperfusion volumes of 44.8 and 310.0 ml, respectively, with a mismatch ratio of 6.9. (A) shows the segmented core and hypoperfusion region in different scenarios with the Dice coefficient between model-predicted and CTP-derived. (B) and (C) display CBF maps for various scenarios. Generally, as the time interval from the peak frames increased, the accuracy of the model decreased.

Table 2 presents the quantitative results and the volume agreement between different mCTP- and CTP-derived images. For CBF map prediction, the model achieved mean values of NRMSE < 0.030, SSIM > 0.950, and LPIPS < 0.070. For *T*_max_ map prediction, the model achieved mean values of NRMSE < 0.055, SSIM > 0.910, and LPIPS < 0.150, indicating favorable quantitative results. For volume agreement, it shows that core volumes (obtained using the threshold of rCBF < 30%) had strong correlation for all groups, with all *P* < 0.001. Volume consistency was good for Pre-1 and Late-1, and moderate for Pre-2 and Late-2. Hypoperfusion volumes (obtained using the threshold of *T*_max_ > 6 s) showed strong correlation for Peak and Late-1, moderate correlation for Pre-1 and Late-2, and weak correlation for Pre-2, with all *P*≤0.001. Figure 4 displays the visual performance of the model across different groups for a patient with moderate infarct size and extensive hypoperfusion. The time interval for advanced or delayed from the peak frames were 2 s for Pre-1 and Late-1, and 4 s for Pre-2 and Late-2. Figure 4A shows the segmented core and hypoperfusion regions with the Dice coefficient between the predicted and labeled areas. Figure 4B and C display the CBF and *T*_max_ derived from different groups and their label maps. Overall, the model demonstrated the capability to advance or delay predictions by up to 2 s for CBF and *T*_max_.

### Comparison between the GAN-BPM and bSVD models

The block-circulant singular value decomposition (bSVD) model yielded metrics of NRMSE 0.074 ± 0.003, SSIM 0.905 ± 0.003, and LPIPS 0.065 ± 0.003 for CBF map prediction, and NRMSE 0.182 ± 0.030, SSIM 0.871 ± 0.002, and LPIPS 0.176 ± 0.002 for the *T*_max_ map prediction. Across the entire testing dataset, the volume agreement between bSVD- and CTP-derived infarct and hypoperfusion tissue was 0.586 (*P* < 0.001) and 0.091 (*P* = 0.361). The mean volume difference for the core between the bSVD-derived (median, 3.325 ml; IQR, 0.698 to 7.202) and CTP-derived was 8.110 ml (LoA, −20.666 to 36.887). The mean volume difference for the hypoperfusion region between the bSVD-derived (median, 118.707 ml; IQR, 38.717 to 446.274) and CTP-derived was −160.844 ml (LoA, −722.399 to 400.711). The blood flow parameter maps calculated directly by the bSVD model tend to overestimate the volume of ischemic tissue compared to those generated by the GAN-BPM model.

### Model performance on the external testing datasets

For the model performance on the FAHXJNU dataset, the mean volume difference for the core between the mCTP-derived (median, 19.8 ml; IQR, 11.1 to 31.5) and CTP-derived (median, 21.8 ml; IQR, 11.5 to 31.5) was −0.817 ml (LoA, −27.2 to 25.6). The mean volume difference for the hypoperfusion between the mCTP-derived (median, 132.0 ml; IQR, 94.8 to 186.7) and CTP-derived (median, 150.2 ml; IQR, 103.5 to 221.8) was 26.8 ml (LoA, −117.3 to 170.9). The quantitative results shown in Table 3 are satisfactory. Volume consistency was good for core volumes and moderate for hypoperfusion volumes. In terms of volume agreement, core volumes showed excellent correlation, while hypoperfusion volumes exhibited moderate correlation (all *P* values < 0.001).

**Table 3.** Quantitative metrics and volumes agreement in the 2 external datasets

	Metrics	FAHXJU	SHSMU
CBF	NRMSE	0.019±1.106e−4	0.108±0.048
	SSIM	0.971±5.234e−04	0.921±0.002
	LPIPS	0.039±8.178e−04	0.088±0.002
*T*_max_	NRMSE	0.058±2.146e−4	0.064±4.596e−4
	SSIM	0.883±9.637e−4	0.817±0.002
	LPIPS	0.141±6.303e−4	0.146±7.009e−4
rCBF < 30%	SCC	0.808	0.720
	ICC	0.883 (0.800, 0.932)	0.731 (0.634, 0.806)
*T*_max_ > 6 s	SCC	0.533	0.540
	ICC	0.635 (0.512, 0.733)	0.499 (0.348, 0.624)

NRMSE, normalized root mean squared error; SSIM, structural similarity index measure; LPIPS, learned perceptual image patch similarity; ICC, intraclass correlation coefficient; SCC, Spearman correlation coefficient.

For the SHSMU dataset, the mean volume difference for the core between the mCTP-derived (median, 35.5 ml; IQR, 22.6 to 55.7) and CTP-derived (median, 39.8 ml; IQR, 33.3 to 46.0) was −7.6 ml (LoA, −74.0 to 58.8). The mean volume difference for hypoperfusion between the mCTP-derived (median, 98.5 ml; IQR, 70.7 to 131.1) and CTP-derived (median, 373.6 ml; IQR, 314.4 to 436.5) was 258.2 ml (LoA, 63.2 to 453.2; *P* < 0.05). The quantitative results shown in Table 3 are also promising. Volume consistency was moderate for core volumes and poor for hypoperfusion volumes. In terms of volume agreement, core volumes exhibited strong correlation, while hypoperfusion volumes showed moderate correlation (all *P* values < 0.001).

In the ISLES2018 dataset, the Dice coefficients for infarct regions predicted by the model and those derived from CTP, compared to the reference standard (follow-up infarct regions manually segmented on DWI images), were 0.239 ± 0.041 and 0.294 ± 0.03, respectively. The model showed similar accuracy in predicting follow-up infarct regions using the full sequence of CTP images.

### Qualitative image analysis

In the Supplementary Materials, Tables S2 and S2 summarize the visual assessment results conducted by 2 radiologists on 102 cases from the FAHJNU dataset and 12 cases from the Sun Yat-sen Memorial Hospital (SYMH) validation dataset. For the FAHJNU dataset, both the mCTP- and CTP-derived maps were deemed diagnostically valuable. The predicted CBF maps demonstrated diagnostic quality comparable to the CTP-derived maps, while the predicted *T*_max_ maps closely aligned with the ground truth. The average scores for both map types exceeded 2.7, reflecting consistently high diagnostic quality. Similarly, for the SYMH dataset, the validation results achieved comparable diagnostic effectiveness to those of the FAHJNU dataset, further supporting the robustness of the proposed approach.

## Discussion

We developed and evaluated a GAN-based model that utilizes spatiotemporal information in mCTP to generate CBF and *T*_max_ maps, eliminating the need for a deconvolution procedure in AIS. Overall, the model demonstrated strong performance in predicting perfusion maps, as well as identifying infarct core and hypoperfused tissue. This model has the potential to assist physicians in making informed clinical decisions for AIS treatment while also reducing radiation exposure.

Current stroke imaging protocols using CT include non-contrast CT (NCCT), CTP, and CTA [19]. NCCT, which is highly sensitive for detecting intracranial hemorrhage, is the first-line imaging modality for stroke assessment. CTA allows for rapid assessment and diagnosis of neuro-vascular pathologies, while CTP, commonly used in stroke centers, offers quantitative assessment of cerebral hemodynamics through perfusion maps generated by deconvolution or non-deconvolution algorithms, aiding in the identification of infarct and hypoperfusion areas. The perfusion maps can be used to guide revascularization treatments. Nevertheless, CTP has several drawbacks: (a) the need for multiple volumetric scans results in high radiation doses; (b) it is sensitive to patient movement; (c) its time–density curves (TDCs) are susceptible to noise; and (d) its widespread use is limited. In contrast, the three-phase mCTP provides hemodynamic information with lower temporal resolution, reducing the overall radiation dose to 15.8% of that used in CTP. Additionally, mCTP is less affected by patient movement and requires less contrast agent compared to mCTA.

Using deep learning to directly predict perfusion maps from mCTP eliminates the need for deconvolution or non-deconvolution algorithms. Deconvolution is crucial in current CTP processing but introduces substantial computational complexity due to physiological changes in arterial contrast delivery, collateral blood flow, and venous outflow components. Additionally, the TDC is sensitive to noise, making it prone to deviations in results. Soltanpour et al. [9] proposed an LSTM-GAN model to generate blood flow parameter maps from CT perfusion sequences that are downsampled at equal intervals along the time axis. They examined the model’s performance using 3 to 23 time points, with sampling done at equal intervals around the peak time of the AIF curve. However, this approach has limited clinical feasibility. In contrast, our work proposes specific sampling time points based on the mCTA scanning protocol, which can be easily implemented in clinical settings. Accordingly, we also investigated possible time-point sampling combinations that may occur in practical acquisition scenarios (see Table 2 and Fig. 4). Additionally, in contrast to the restricted application range of CTP, mCTA, which adds 2 phases to CTA, has a wider application range [11]. Previous studies [13,14] have used combinations of NCCT and mCTA to predict perfusion maps or assess hypoperfused tissue. For example, Qiu et al. [14] developed three random forest models trained using final segmented infarct mask images and hypoperfused areas calculated from *T*_max_ maps, employing a method similar to image segmentation tasks to predict cerebral tissue perfusion outcomes. Benali et al. [13] validated the StrokeSENS software, which generates perfusion maps from NCCT and mCTA volumes. The authors found that the perfusion maps produced by this software performed similarly to those generated by CTP, which uses NCCT and mCTA volumes to calculate *T*_max_ and CBF with a non-deconvolution algorithm, incorporating these maps into a neural network to generate *T*_max_ and relative CBF. These studies suggest that NCCT and mCTA can be used for perfusion maps, though high registration accuracy is needed due to differences in scanning parameters such as slice thickness, tube voltage, and current settings. Directly using mCTP for prediction provides 2 main advantages: (a) it directly generates perfusion maps for easy observation of the occlusion site and status, and (b) it avoids the additional computational complexity introduced by deconvolution or non-deconvolution algorithms. Moreover, deep learning techniques have been successfully applied to directly predict perfusion maps. For example, some studies have shown this approach with CTP sequence images [9,20], NCCT [21], and non-perfusion mismatch ratio (MR) modalities [22,23]. These studies have established the feasibility and potential of using deep learning models to predict perfusion maps from mCTP.

In this present study, we developed a GAN-based model to estimate perfusion parameters, specifically CBF and *T*_max_, achieving promising results. The model effectively predicted mCTP perfusion maps using the arterial and venous peak frames along with delayed frames, and delivered satisfactory quantitative results. The correlation between the overall lesion volume using a *T*_max_ > 6 s threshold was strong compared to the reference, while the volume obtained using an rCBF < 30% threshold showed a high correlation with the reference. The volume consistency also showed the prediction reliability of our model. The model also successfully identified ischemic and infarct lesions of various sizes, although prediction accuracy decreased with larger lesion sizes. In analyzing different phase selections, we found that prediction accuracy decreased with increasing time intervals. The deep learning model still produced acceptable results when the phase selection was advanced or delayed by up to 2 s. As the time interval increased, the arterial and venous visualization in the images decreased, causing the model to rely on peak-phase data for earlier phases, potentially underestimating *T*_max_ maps.

Similarly, delayed phase selection led to venous reflux, resulting in the model applying peak phases data to later phases, which may have led to an underestimation of hypoperfused and infarct regions. Overall, the model’s performance in predicting *T*_max_ parameter maps was inferior to its performance for CBF maps. This discrepancy may be due to the sparse time values in *T*_max_ maps, which differed markedly from CT image data distributions, making it challenging for the model to learn an accurate mapping. Additionally, noise and minor artifacts also affected the model’s prediction performance. Compared to the dSVD model, our model improves the prediction of blood flow parameter maps using mCTP. The prediction of infarct core volumes is reflected in the performance of the CBF parameter map, where the bSVD model produces results close to ours. However, the *T*_max_ parameter map, which is sensitive to time parameters, shows that the bSVD model’s estimation of hypoperfusion volumes is markedly inferior to that of our model. For the external testing, the model performed similar results on the FAHXJU and SHSMU datasets on CBF maps prediction, and good results on *T*_max_ map predictions of FAHXJU but weaker results for SHSMU. The differences may be caused by different frame scanning settings. The scanning frames of the SHSMU dataset is 18 with fixed time interval, while it is 19 with different time intervals for the FAHJNU and FAHXJU datasets. Moreover, the onset-to-examination time of the SHSMU dataset was generally longer than others. For the ISLES2018 dataset, the parameter maps derived from the model showed performance comparable to those from CTP deconvolution when compared to follow-up DWI-defined infarct regions. However, all methods showed weaker overlap with the standard DWI-defined infarct regions (with Dice coefficients < 30%). Further analysis revealed potential reasons: (a) the external dataset consisted of multicenter data with unknown CT machine types and scanning protocols, possibly leading to distribution bias compared to the internal dataset; (b) national population differences might introduce subtle deviations; and (c) inherent limitations in registration between DWI and CT imaging modalities.

In the qualitative image analysis, our mCTP approach produced diagnostic results that were virtually identical to the parameter maps derived from conventional CTP, highlighting the practical feasibility of the method. Notably, the predicted CBF images showed a slight improvement in quality over the ground-truth labels.

This study has some limitations. First, the model did not account for the complexities in real scans, such as artifacts or noise levels affecting data distribution. Future improvements could come from incorporating a more diverse range of data to improve the model’s generalizability. Moreover, determining stroke lesion size and formulating treatment plans necessitate a thorough clinical assessment, including Alberta Stroke Program Early CT Score, collateral circulation assessment, and time from onset to perfusion, and various pathophysiological factors such as cerebral autoregulation and tissue tolerance to ischemia and hypoxia [24–26]. The model only utilized the images information and did not fully utilize other clinical data. Therefore, future developments could focus on 2 areas: (a) incorporating additional clinical data to the model; and (b) involving experts to delineate low perfusion and infarct regions from *T*_max_ and rCBF images, which could then be used to create a new loss function or introduce actual scan time as a variable in the model’s training process.

In conclusion, we developed a GAN-BMP model to predict CBF and *T*_max_ maps using mCTP in AIS patients. The quantitative and volumetric agreement results from the datasets show the model’s prominent potential as a novel diagnostic tool for predicting perfusion maps from mCTP, while also effectively reducing the radiation dose associated with perfusion imaging.

## Materials and Methods

### Patients and datasets

The retrospective cohort study was approved by the Ethics Committee of the hospital, and the requirement for informed consent was waived. The dataset for model training and internal testing was collected from FAHJNU. The FAHJNU dataset included 662 patients with AIS diagnosed between December 2022 and May 2024. All patients underwent CTP scans (NeuViz Epoch; Neusoft Medical Systems, Shenyang, China). The CTP scanning protocol was as follows: a tube voltage of 80 kVp, a tube current-time product of 250 mAs, a slice thickness of 5 mm, and 19 frames each slice, resulting in a dose-length product of 2,991.12mGy∙cm (range, 160 mm) with a total exposure time of 14.25 s. Perfusion maps, such as CBF and *T*_max_, were calculated using the bSVD method with an AIF selected from the middle cerebral artery region [27]. Additionally, the CTP sequences were interpolated to 0.5-s intervals. Two thresholds of *T*_max_ > 6 s and rCBF < 30% were utilized to segment hypoperfused and core tissues [28,29]. The bSVD algorithm core is publicly available at https://github.com/ruogufang/pct.

Three external testing datasets were utilized in this study. The first dataset, referred to as FAHXJU, was obtained from the First Affiliated Hospital of Xi’an Jiaotong University. It includes 48 patients undergoing CTP scans (Revolution CT; GE Medical Systems, Chicago, America) with 768 slices, and 19 frames per case, collected between January 2023 and November 2023. The CTP acquisition parameters were as follows: a tube voltage of 80 kVp, a tube current of 75 mA, and a slice thickness of 5 mm. The second dataset, named SHSMU, was collected from Shunde Hospital of Southern Medical University. This dataset consists of 115 patients undergoing CTP scans (IQon-Spectral CT; Philips, Amsterdam, Netherlands) with 1,840 slices, and 18 frames per case, collected between October 2022 and October 2024. The CTP acquisition parameters were as follows: a tube voltage of 80 kVp, a tube current of 80 mA, and a slice thickness of 5 mm. Perfusion maps for both datasets were calculated using the bSVD method. Additionally, the public ISLES2018 dataset [30,31], acquired from three U.S. centers and one Australian center, was used as the third external testing set. This dataset includes 156 CTP acquisitions from 103 AIS patients, and includes the following imaging modalities: baseline NCCT, CTP, and 4 types of perfusion maps (CBF, CBV, MTT, and *T*_max_) generated by an automated software (Rapid; iSchemaview, Menlo Park, CA), and infarct core masks segmented from DWI acquired within 3 h post-CTP. Furthermore, regions of interest used for estimating the AIF and VOF from this dataset, as identified in a previous study [32], were utilized to generate the mCTP images. The ISLES2018 dataset is publicly available at http://www.isles-challenge.org/ISLES2018/.

To further validate the feasibility of the proposed mCTP approach in reducing radiation dose for CTP imaging, we assembled a dataset comprising 12 patients from Sun Yat-sen Memorial Hospital, referred to as the SYMH dataset. This dataset includes paired mCTA and corresponding CTP images acquired using a SOMATOM Force scanner (Siemens, Munich, Germany). The mCTA scanning parameters were as follows: a tube voltage of 110 kVp, a tube current of 258 mA, and a slice thickness of 1 mm. The CTP scanning parameters were as follows: a tube voltage of 80 kVp, a tube current-time product of 247 mA, a slice thickness of 5 mm, and 25 frames each slice.

**Fig. 5.**
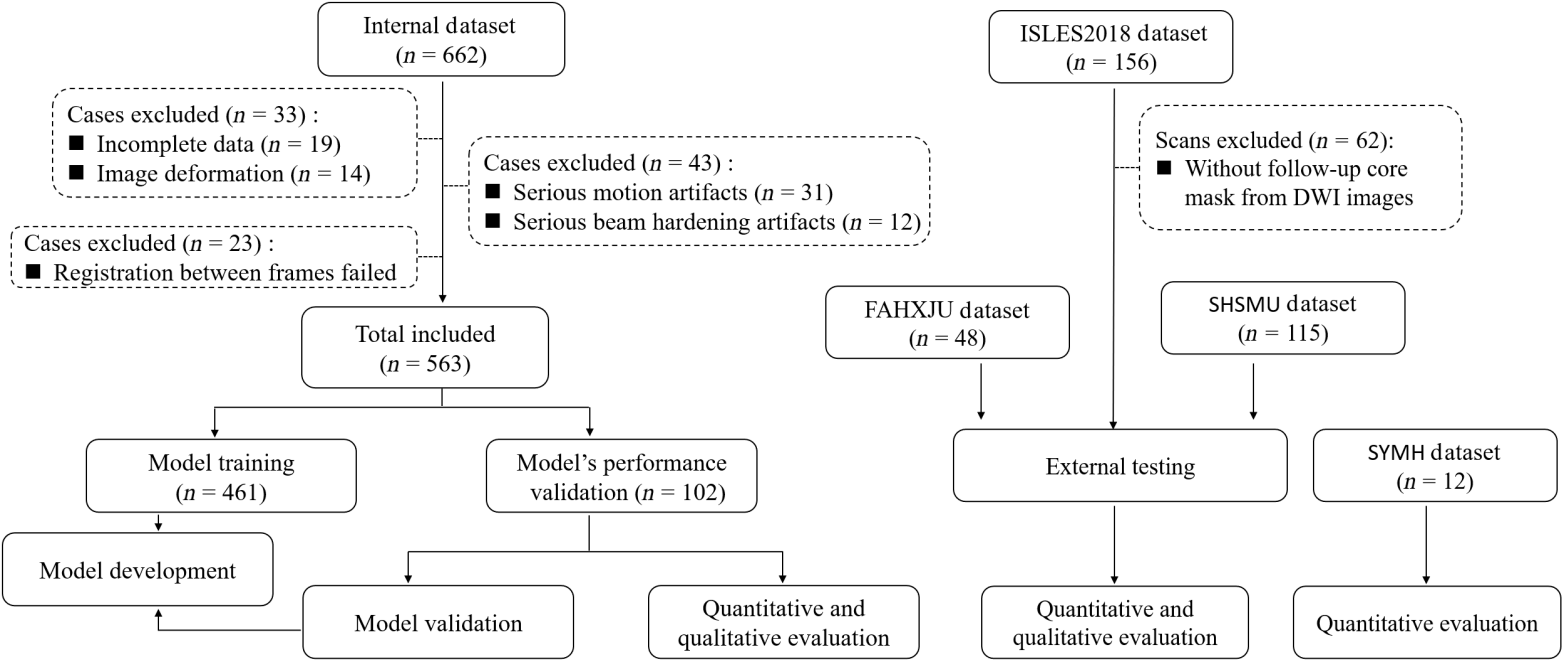
Flowchart of patient inclusion for model training and testing.

After excluding cases with poor image quality or registration failures, the FAHXJU dataset was randomly split into a training cohort (*n* = 461) for model development and an internal testing cohort (*n* = 102) for model validation and evaluation. The FAHXJU and SHSMU datasets were all utilized for external testing. For the ISLES2018 dataset, we used images from cases with follow-up infarct core masks derived from DWI images (*n* = 94,287 images) to further assess the model’s generalization performance. A flowchart illustrating the patient selection process from the 2 datasets is presented in Fig. 5.

### mCTP acquisition and data preprocessing

The mCTA scanning protocol utilized a three-phase dynamic acquisition technique [11]: the first phase (peak arterial, i.e., head and neck CTA) was acquired from arch to vertex with a multirow detector CT, which was timed and triggered by bolus monitoring; the second (peak venous) and third phases (late venous) both covered the skull base to vertex. The first phase was mostly acquired in less than 7 s, while the other phases were acquired after a delay of 4 s from the previous phase, allowing for table positioning to the skull base. Similar to the sampling approach used in mCTA, mCTP consists of three phases of CTP images: the peak frames of AIF, the peak frame of VOF, and a delay frame following VOF peak frame (with an 8-s interval) [11]. Additionally, a comparison of the scanning protocols for mCTA, mCTP, and CTP is provided in Table S1. We also investigated how the selection of phases in mCTP affects the accuracy of perfusion maps. We adjusted the phase selection by either advancing or delaying the frames by 1 or 2 frames compared to the ideal mCTP phase selection.

For data preprocessing, we performed frame-to-frame registration on each image layer using ANTSPYX software (v0.4.2) and applied Gaussian filters for spatial and temporal filtering both at the image level and along the time axis with a kernel size of 7 and 3. The registration operation employed rigid transformation with mutual Information as the similarity metric, optimized through a gradient descent algorithm integrated with a three-level multiresolution pyramid. Registration is important for model learning as perfusion maps are calculated at the pixel level. Additionally, we used a threshold range of 10 to 80 HU (Hounsfield unit) to segment the brain, and normalized the input and label images to the range [−1,1].

### GAN-BPM model development

The GAN-BPM model, which includes a generator with a 3-dimensional (3D) convolution-based block and a Unet-based 2D convolution network, as well as a PatchGAN discriminator, was selected as the backbone network to generate the perfusion maps from mCTP. The network processed a 3D dataset with a size of N×N×D, where *N* represents the matrix size (*N* = 512) and *D* denotes the channel depth (*D* = 3, corresponding to the three phases of mCTP). The 3D convolution block extracted temporal information, while the 2D convolutional network captured spatial details. The generator and discriminator trained adversarially to calculate accurate perfusion maps (CBF or *T*_max_). The total loss function of generator, detailed in the Supplementary Materials, includes pixel loss, adversarial loss, and an extreme loss that directs the model to focus on infarct and high blood flow regions during training.

Model weights were initialized randomly and optimized using the Adam algorithm ($\beta_{1}=0.500,\beta_{2}=0.999$) with a batch size of 15. The learning rates for both the generator and discriminator was set to $1e^{-4}$, and the batch size was 15. The training epochs were 80 for the *T*_max_-predicted model and 85 for the CBF-predicted model. All experiments were done on NVIDIA RTX A6000 GPUs using Pytorch 2.1.1.

### Performance evaluation

The model’s performance was evaluated using both quantitative and subjective assessments. In terms of quantitative assessment, the NRMSE, SSIM, and LPIPS were leveraged to measure the similarity between the predicted perfusion maps and those derived from the CTP images. NRMSE assesses the average squared discrepancy between the predicted and the reference images, normalized by the data range, with lower values indicating higher accuracy and consistency. SSIM assesses the perceptual quality by evaluating luminance, contrast, and structural similarity, with higher values reflecting better visual quality and closer resemblance to the reference image. LPIPS measures the perceptual similarity by comparing deep features from a neural network, with lower scores indicating better perceptual fidelity and closer alignment with the reference.

To evaluate the agreement between the actual and generated perfusion maps, we compared the infarct volume (threshold: rCBF < 30%) and hypoperfused volume (threshold: *T*_max_ > 6 s) between predicted and labeled maps for each case in the external testing set. To further evaluate the model’s improvement in predicting blood flow parameter maps using mCTP, we compared its performance with the conventional deconvolution algorithm. Specifically, we combined the mCTP images with the first unenhanced frame to form 4 phases. Linear interpolation was then applied across the time frames to complete the sequence, and the bSVD model was used to calculate the CBF and *T*_max_ parameter maps. Additionally, we performed threshold-based segmentation on the predicted and standard parameter maps using rCBF < 30% to identify infarct regions in the ISLES2018 dataset. These regions were then compared with manually segmented infarct regions on DWI images using the Dice coefficient to evaluate the spatial agreement with the standard infarct regions.

### Qualitative image analysis

Image quality assessment was conducted independently by 2 board-certified neuroradiologists with 5 and 8 years of experience, respectively. Both reviewers were blinded to patient information and the source of the perfusion maps (i.e., whether they were model-derived or CTP-derived). The evaluation was performed on the testing set from the FAHJNU and SYMH datasets. Diagnostic confidence was assessed using a 3-point Likert scale [33]: 1 = poor, non-diagnostic; 2 = moderate, diagnostic; 3 = good, high diagnostic confidence.

### Statistical analysis

Bland–Altman plots were utilized to illustrate mean differences and LoA between mCTP-predicted and CTP reference volumes. Scatter plots were employed to visualize the volume relationship between mCTP and CTP. The intraclass correlation coefficient (ICC) and the SCC were used to describe the volume consistency and agreement. The ICC values were interpreted based on the suggested criteria: <0.50, poor; 0.50 to 0.75, moderate; 0.75 to 0.90, good; and >0.90, excellent. SCCs were classified as follows: >0.80 as excellent, 0.60 to 0.79 as strong, 0.40 to 0.59 as moderate, and <0.40 as weak. A 2-tailed *P* < 0.05 was considered significant. All statistical analyses were performed using Origin 2024 (OriginLab Corporation, Northampton, MA, USA) and PyCharm 2022.2.3 (JetBrains, Prague, Czech Republic).

## Data Availability

The data that support the findings of this study are available from the corresponding authors, depending on reasonable request from qualified investigators after permission of the appropriate regulatory bodies.
