# Supplementary material for: Perfusion Parameter Map Generation from 3 Phases of Computed Tomography Perfusion in Stroke Using Generative Adversarial Networks
Source: Research (Wash D C). 2025 Apr 30;8:0689. doi: 10.34133/research.0689 (PMC12041647; doi:10.34133/research.0689)
Supplement: Supplementary 1 — Supplementary Methods Fig. S1 Tables S1 to S3 [file research.0689.f1.docx]

**SUPPLEMENTAL MATERIAL**

**Supplemental Methods**

**1. Network architecture**

The GAN-BPM model includes a generator with a 3D convolution-based block and a Unet-based 2D convolution networks (shown in Figure S1), as well as a PatchGAN discriminator. The 3D convolution-based block consists of two 3D convolutional operations with a $3\times1\times1$ kernel and a $2\times1\times1$ stride, extracting related temporal information along time axis. Encoder structure of the Unet-based 2D convolution networks includes six 2D convolutional operations with a $4\times4$ kernel and a $2\times2$ stride, capturing spatial details of images. Then, the decoder structure, containing seven up-sampling sup-blocks (consists of a unsample layers, a 2D reflection padding layer, and a 2D convolution layer with a $3\times3$ kernel and a $2\times2$ stride), maps the latent space to the target space.

The discriminator contains four downsampling convolutional blocks with a $4\times4$ kernel and a $2\times2$ stride, and one output convolutional block with a $4\times4$ kernel and a $1\times1$ stride (two convolutional layers). Before final output, the output of convolutional block was input to a sigmoid function.


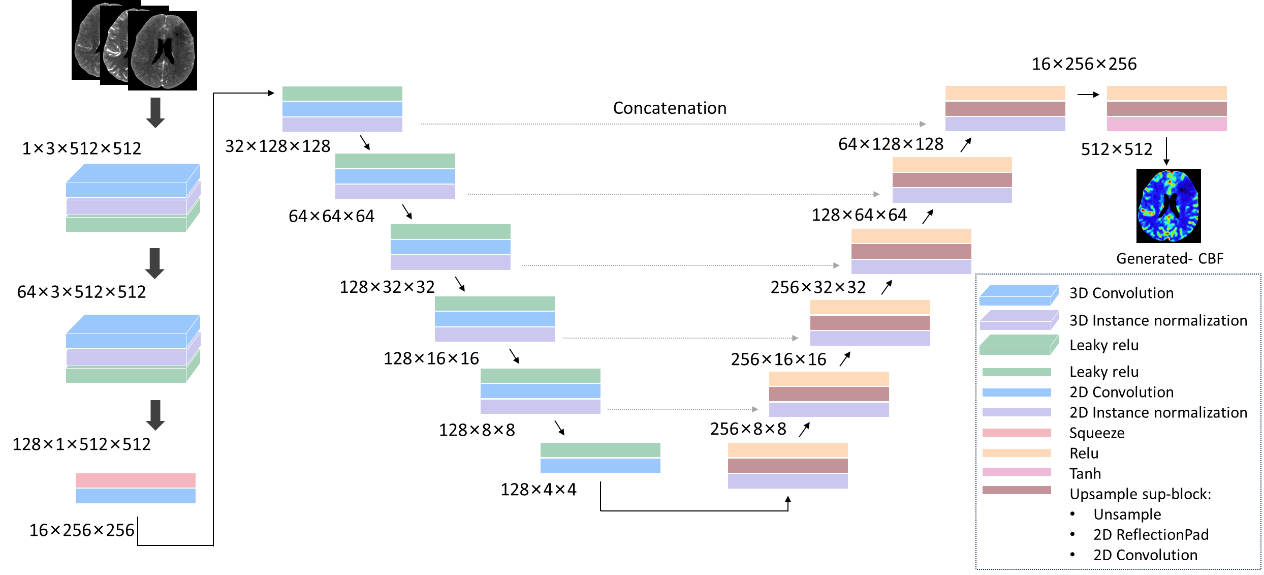


**Figure S1. The generative adversarial network-based brain perfusion map synthetic (GAN-BPM) model generator structure.**

**2. Loss functions**

The generator and discriminator learn against each other to produce accurate perfusion maps. The total loss function of generator $L_{total}$ shown in Equation (1), include pixel loss $L_{pix}$, adversarial loss $L_{adv}$, and extreme loss $L_{ext}$ which is utilized to remind the model to posse attention on infarct region and high blood flow region during learning. $\lambda_{1}$, $\lambda_{2}$ and $\lambda_{3}$ are the weight parameters, setting as 100, 1 and 50 here.

$L_{total}={\lambda_{1}*L}_{pix}+{\lambda_{2}*L}_{adv}+{\lambda_{3}*L}_{ext},$ (1)

$L_{adv}=\frac{1}{2}E_{A\in p_{data}(A)}\left[ {\left| |D\left( G\left( A \right),A \right)-1 \right||}_{2} \right],$ (2)

$L_{pix}=E_{A\in p_{data}\left( A \right),B\in p_{data}\left( B \right)}\left[ {\left| |B-G\left( A \right) \right||}_{1} \right],$ (3)

$L_{ext}=E_{A\in p_{data}\left( A \right),B\in p_{data}\left( B \right)}\left[ {W_{B}\cdot\left| |B-G\left( A \right) \right||}_{2} \right],$ (4)

where A denotes the input volumes, B indicates the label images (CBF or Tmax map), and $W_{B}$ is a weight image constructed by label image ($W_{B}=B\odot B$, $\odot$ is an ​element-wise product). $G\left( \cdot\right)$ represents the generator and $D\left( \cdot\right)$ expresses the discriminator.

The discriminator loss is described in Equation (5).

$L_{D}=\frac{1}{2}E_{A\in p_{data}\left( A \right),B\in p_{data}\left( B \right)}\left[ {\left| |D(B, A)-1 \right||}_{2} \right]+\frac{1}{2}E_{A\in p_{data}(A)}\left[ {\left| |D\left( G\left( A \right),A \right) \right||}_{2} \right].$ (5)

**Supplemental Scanning protocol**

Table S1. A case of comparison between mCTA, mCTP, and CTP scanning protocols.

|  | mCTA | mCTP | CTP |
| --- | --- | --- | --- |
| Scanning time points | peak arterial, peak venous, late venous^1^ | peak arterial, peak venous, late venous | 28 passes over 66 seconds^2^ |
| Frame count | 3^2^ | 3 | 28^2^ |
| Contrast usage (mL) | 80^2^ | $\leq$40^*^ | 40^2^ |
| Contrast material | Optiray 320; Mallinckrodt Pharmaceuticals, St. Louis, MO^2^ | Optiray 320; Mallinckrodt Pharmaceuticals, St. Louis, MO^2^ | Optiray 320; Mallinckrodt Pharmaceuticals, St. Louis, MO^2^ |
| Tube voltage (kVp) | 120^2^ | 80 | 80^2^ |
| Tube current (mA) | 130^2^ | 190 | 190^2^ |

Note: The parameters presented in the table was a case of our mCTP protocol which was referred from the previous research^1^. * suggested that the contrast agent usage of mCTP is less than usage of CTP for CTP requires a sufficient contrast dose to maintain enhancement during the equilibrium period^2^.

**Supplemental Results**

Table S2. Diagnostic quality evaluation on FAHJNU dataset with 102 cases.

|  | Doctor1 | | | Doctor2 | | |
| --- | --- | --- | --- | --- | --- | --- |
| Score | predicted | label | P | predicted | label | P |
| CBF | $2.902\pm0.088$ | $2.873\pm0.111$ | 0.441 | $2.823\pm0.145$ | $2.824\pm0.145$ | 1.000 |
|  | 90.2% | 87.3% |  | 82.4% | 82.4% |  |
| Tmax | $2.853\pm0.125$ | $2.882\pm0.104$ | 0.494 | $2.784\pm0.169$ | $2.804\pm0.158$ | 0.620 |
|  | 85.3% | 88.2% |  | 78.4% | 80.4% |  |

Note: Image quality is defined as: 1= poor and non-diagnostic; 2= moderate and diagnostic; 3=good and good diagnostic confidence. For each type of maps, we presented the scores (mean ± standard deviation ) and the percentage of which equal to 3. P<0.05 is considered to be significantly different.

Table S3. Diagnostic quality evaluation on SYMH dataset with 12 cases.

| Doctor1 | | | | Doctor2 | | | |
| --- | --- | --- | --- | --- | --- | --- | --- |
| CBF | | Tmax | | CBF | | Tmax | |
| predicted | label | predicted | label | predicted | label | predicted | label |
| 3 | 2 | 2 | 2 | 2 | 2 | 3 | 2 |
| 3 | 2 | 2 | 2 | 3 | 2 | 2 | 2 |
| 3 | 3 | 2 | 3 | 3 | 3 | 2 | 2 |
| 3 | 3 | 2 | 2 | 3 | 2 | 3 | 2 |
| 3 | 3 | 3 | 3 | 3 | 3 | 3 | 3 |
| 3 | 3 | 3 | 3 | 3 | 2 | 3 | 3 |
| 3 | 2 | 3 | 2 | 2 | 2 | 3 | 2 |
| 3 | 3 | 3 | 3 | 3 | 3 | 3 | 3 |
| 3 | 2 | 3 | 3 | 3 | 2 | 3 | 3 |
| 3 | 3 | 3 | 3 | 3 | 3 | 3 | 3 |
| 3 | 3 | 3 | 3 | 3 | 3 | 3 | 3 |
| 3 | 3 | 3 | 3 | 3 | 3 | 3 | 2 |
| 100% | 66.7% | 66.7% | 66.7% | 83.3% | 50% | 83.3% | 50% |

Note: For each type of maps, we presented the scores from the 12 cases and the percentage of which equal to 3.

**Supplemental References**

1. Chung, K.J., Pandey, S.K., Khaw, A.V., Lee, T.Y. Multiphase CT angiography perfusion maps for predicting target mismatch and ischemic lesion volumes. Sci Rep. 13, 21976 (2023).
2. Wintermark, M. et al. Dynamic perfusion CT: optimizing the temporal resolution and contrast volume for calculation of perfusion CT parameters in stroke patients. Am J Neuroradiol. 25, 720-729 (2004).
